# Cyclic Oligosaccharide-Induced Modulation of Immunoglobulin A Reactivity to Gut Bacteria Contributes to Alterations in the Bacterial Community Structure

**DOI:** 10.3390/nu16172824

**Published:** 2024-08-23

**Authors:** Taisei Miyamoto, Takeshi Tsuruta, Mao Teraoka, Tianyang Wang, Naoki Nishino

**Affiliations:** Graduate School of Environmental and Life Science, Okayama University, Okayama 700-8530, Japan; miyamoto2030@s.okayama-u.ac.jp (T.M.); mao.teraoka@s.okayama-u.ac.jp (M.T.); pvh11tk5@s.okayama-u.ac.jp (T.W.); j1oufeed@cc.okayama-u.ac.jp (N.N.)

**Keywords:** cyclic oligosaccharides, gut bacteria, immunoglobulin A

## Abstract

Immunoglobulin A (IgA) is a major gut antibody that coats commensal gut bacteria and contributes to shaping a stable gut bacterial composition. Although previous studies have shown that cyclic oligosaccharides, including cyclic nigerosyl-1,6-nigerose (CNN) and cyclodextrins (CDs, including αCD, βCD, and γCD), alter the gut bacterial composition, it remains unclear whether cyclic oligosaccharides modify the IgA coating of gut bacteria, which relates to cyclic oligosaccharide-induced alteration of the gut bacterial composition. To address this issue, mice were maintained for 12 weeks on diets containing CNN, αCD, βCD, or γCD; the animals’ feces were evaluated for their bacterial composition and the IgA coating index (ICI), a measure of the degree of IgA coating of bacteria. We observed that the intake of each cyclic oligosaccharide altered the gut bacterial composition, with changes in the ICI found at both the phylum and genus levels. The ICI for Bacillota, Lachnospiraceae NK4A136 group, UC Lachnospiraceae, and *Tuzzerella* were significantly and positively correlated with the relative abundance (RA) in total bacteria for these bacteria; in contrast, significant correlations were not seen for other phyla and genera. Our observations suggest that cyclic oligosaccharide-induced modulation of the IgA coating of gut bacteria may partly relate to changes in the community structure of the gut bacteria.

## 1. Introduction

Commensal gut bacteria contribute to human health by producing vitamins [1] and bioactive molecules such as sphingolipids [2] and 10-hydroxy-cis-12-octadecenoic acid [3], metabolizing indigestible carbohydrates into short-chain fatty acids (SCFAs) [4], educating the host immune system [5], strengthening gut barrier integrity [6], and serving other functions. The composition of the gut bacteria is known to be modulated by the type of diet [7,8] and specific dietary ingredients such as dietary polyphenols, probiotics [8], and prebiotics, substrates that are selectively utilized by host microorganisms to confer a health benefit [9]. Cyclic oligosaccharides are ring-shaped molecules that consist of various sugar moieties linked together; some of these molecules have been shown to exhibit prebiotic effects. Cyclic nigerosyl-1,6-nigerose (CNN) is a cyclic oligosaccharide consisting of four D-glucopyranosyl residues linked by alternating α-1,3 and α-1,6 glucosidic linkages. We previously demonstrated that the oral administration of CNN induces the amelioration of colonic inflammation in dextran sulfate sodium-induced colitis mice; this effect was accompanied by alterations of the fecal bacterial community, including an accumulation of members of the phylum Bacteroidota and a depletion of members of the phylum Bacillota [10]. Another class of cyclic oligosaccharides is the cyclodextrins (CDs), cyclic polymers consisting of α-1,4-linked D-glucopyranosyl residues. The most common natural CDs are αCD, βCD, and γCD, which contain six, seven, and eight glucose units, respectively. αCD and βCD are able to reach the colon without degradation by digestive enzymes [11]. In contrast, γCD is known to be digested in the small intestine by luminal and epithelial enzymes, although undigested γCD or the hydrolyzed fraction still is able to reach the colon [12]. Zhu et al. demonstrated that, in high-fat diet-fed mice, oral administration of any of these CDs changes the gut bacterial composition (compared to that seen with cellulose), and further showed that the modulatory effects differed among CDs [13].

Previous studies have demonstrated that prebiotics are fermented selectively by saccharolytic bacteria in the colon [14], generating organic acids including lactate, acetate, propionate, and butyrate and moderate decreases in colonic pH [15]. These studies suggest a possible underlying mechanism by which prebiotics change the gut bacterial composition: some species preferentially utilize prebiotics, resulting in a reduction in colonic pH and competitive inhibition of the growth of a subset of the gut bacteria [16]. However, it remains unclear how the gut immune system contributes to prebiotic-induced alterations in gut bacterial composition. Immunoglobulin A (IgA) is the major antibody isotype secreted into the gut [17], where this protein specifically coats gut bacteria [18]. Suzuki et al. showed that IgA suppresses the overgrowth of bacteria in the small intestine [19]. Mirpuri et al. reported that the IgA coating of specific bacteria critically influences the maturation of gut bacteria in newborn mice [20]. Furthermore, using mice deficient for the *Pd1* gene (encoding the programmed cell death 1 protein), Kawamoto et al. demonstrated that a decrease in the IgA coating of gut bacteria alters the gut bacterial composition [21]. Together, these reports suggest that gut IgA contributes to shaping and maintaining a stable gut bacterial composition. We previously observed that oral administration of CNN modulates IgA reactivity to gut bacteria in high-fat diet-fed mice, resulting in a decrease in the IgA coating of Lachnospiraceae and an increase in the IgA coating of Erysipelatoclostridiaceae, Enterobacteriaceae, and Xanthobacteraceae (compared to control animals) [22]. Our observation implies that the IgA coating profile of commensal gut bacteria may be altered by the intake of cyclic oligosaccharides. Therefore, we hypothesized that the change in IgA coating is related to cyclic oligosaccharide-induced changes in the gut bacterial composition. In the present study, we tested this proposal by maintaining mice on diets containing CNN, αCD, βCD, or γCD (or on a control diet), and then collecting fecal samples at the 12-week time point. The fecal samples were assessed for bacterial composition and the IgA coating of bacteria, and potential correlations between these parameters were analyzed.

## 2. Materials and Methods

### 2.1. Dosage Information

The CNN was a gift from Hayashibara Co., Ltd. (Okayama, Japan). αCD, βCD, and γCD were purchased from Kanto Kagaku Co., Ltd. (Tokyo, Japan). Each cyclic oligosaccharide was added to the diet at 50 g/kg by replacing α-maize starch with CNN, αCD, βCD, or γCD. Mice were maintained on the respective diet for 12 weeks; the compositions of the experimental diets are shown in Table 1. The properties of each cyclic oligosaccharide are shown in Table 2.

### 2.2. Animal Experiment

The experimental protocols were approved by the Animal Care and Use Committee of Okayama University, Japan (Approval No. OKU-2022196, Date: 8 April 2022). Thirty BALB/c mice (males, seven weeks old) were purchased from The Jackson Laboratory (Bar Harbor, ME, USA). Throughout the study, animals were housed in a temperature-controlled room with a 12 h/12 h light/dark cycle and were provided with ad libitum access to food and water. Following a 7-day acclimation period, the animals were weighed, randomized, and assigned to five groups of 6 mice, such that the groups had similar mean body weights. The sample size was determined based on a previous study [13]. Following assignment, each group was shifted to the respective experimental diet. During the experimental phase, the mice were housed at 3 per cage, and body weight was measured weekly. The daily feed intake per cage was measured every three weeks using feeding equipment with dome-type covers (Roden CAFE, Oriental Yeast Co., Tokyo, Japan); the resulting data were used to calculate the mean feed intake (per day, per mouse). After the mice had been maintained on the indicated experimental diet for 12 weeks, a fresh fecal sample was collected from each animal and stored at −20 °C pending analysis. The mice were then euthanized by CO_2_ narcosis. To minimize potential confounders, one mouse from each group was dissected in turn. At necropsy, the gut of each mouse was excised, and the cecal content was collected and weighed. A length of approximately 1 cm of the colonic tissue was collected into the RNA extraction reagent ISOGEN II (Nippon Gene, Tokyo, Japan). The remaining colonic tissue was placed on ice pending processing (as described below) for flow cytometric analysis. The remaining tissues were discarded without further examination. The animal study then was repeated, providing samples from two independent experiments comprising 30 mice each; therefore, 60 mice in total were used across the two independent experiments.

### 2.3. 16S rRNA Gene Sequencing of IgA^+^ and IgA^−^ Bacteria

Each fecal pellet was suspended in 500 µL of phosphate-buffered saline (PBS) and subjected to centrifugation (100× *g*, 20 min, 4 °C) to clear the suspension. The resulting supernatant was transferred to a fresh tube and subjected to a second round of centrifugation (9000× *g*, 10 min, 4 °C) to pellet the bacteria. The resulting supernatant was collected for quantification of IgA by enzyme-linked immunosorbent assay (ELISA; see the next section). The resulting bacterial pellet was washed twice with PBS and used for the isolation of IgA-coated bacteria (IgA^+^ bacteria) and non-coated bacteria (IgA^−^ bacteria). IgA^+^ and IgA^−^ bacteria were isolated by an affinity purification method using magnetic beads, as previously reported [22]. DNA was extracted from the IgA^+^ and IgA^−^ bacteria using the Nucleospin Tissue XS kit (Macherey-Nagel, Duren, Germany) according to the manufacturer’s instructions. PCR amplification of bacterial 16S rRNA genes and purification of the PCR amplicons were performed as described previously [23]. The purified amplicons were subjected to paired-end sequencing (2 × 250 b) on an Illumina MiSeq platform (Illumina, San Diego, CA, USA); sequencing was conducted at FASMAC Co., Ltd. (Kanagawa, Japan). The archived raw sequences were processed using the Quantitative Insights Into Microbial Ecology 2 (QIIME2, version 2021.11) software package [24]. The raw sequences were filtered to remove reads at any sites receiving a quality score < 25 and length < 135 b. Sequences were joined by paired ends, and chimeric sequences were identified and removed using the dada2 package executed in the R software (version 4.1.2) [25,26]. High-quality and non-chimeric sequences were grouped into sequence variants. Taxonomy was assigned using the SILVA rRNA database, and bacterial clustering was analyzed at the phylum and genus levels [27]. Data were normalized using “qiime diversity alpha-rarefaction”. Relative abundances (RAs, expressed as percentages) at the phylum and genus levels were calculated (separately) for IgA^+^ and IgA^−^ bacteria. To evaluate the degree of IgA coating of bacteria in each phylum and genus, the IgA coating index (ICI) was calculated as defined previously [28]. In short, the ICI was calculated by dividing the RA of IgA^+^ bacteria by the RA of IgA^−^ bacteria. When the RA of IgA^−^ bacteria was zero, the lowest value (0.001%) was substituted. When both of the RAs were zero, the ICI was judged as not detected (ND), given that this ratio was not calculable.

### 2.4. 16S rRNA Gene Sequencing of Total Bacteria in Feces

Total bacterial DNA was extracted from the feces using a QIAamp Fast DNA Stool Mini Kit (QIAGEN, Tokyo, Japan) according to the manufacturer’s instructions. PCR amplification of 16S rRNA genes, purification of the PCR amplicons, sequencing using an Illumina MiSeq, and QIIME2 analysis were conducted using a workflow like that described in Section 2.3. RAs (%) in total bacteria were calculated at the phylum and genus levels. The α-diversity analysis, including Pielou’s evenness index and the Shannon diversity index, was conducted. The similarities among microbial communities in different samples were determined using principal coordinate analysis based on Unweighted UniFrac distance.

### 2.5. Quantification of Fecal IgA Concentration and Flow Cytometric Analysis of Colonic IgA-Secreting Plasma Cells (PCs)

The concentration of IgA in the fecal supernatant was measured by ELISA using anti-mouse IgA antibody (BioLegend, San Diego, CA, USA), horse radish peroxidase (HRP)-conjugated anti-mouse IgA antibody (SouthernBiotech, Birmingham, AL, USA), and mouse reference serum (Bethyl Laboratories, Montgomery, TX, USA) according to the respective manufacturers’ instructions.

The proportion of IgA-secreting PCs (IgA-PCs) in the colon was evaluated as previously described [29], with some modifications. In brief, a portion of the colonic tissue that had been stored on ice was minced and subjected to two 20 min rounds of stirring at 37 °C in calcium/magnesium-free Hanks’ Balanced Salt Solution (HBSS, Nacalai tesque, Kyoto, Japan) containing 1 mM ethylenediaminetetraacetic acid (EDTA) (Nacalai tesque). The minced tissue was washed three times with PBS before being subjected to digestion (30 min, 37 °C) in calcium/magnesium-containing HBSS supplemented with 1.5 mg/mL collagenase (Fujifilm Wako Chemicals, Tokyo, Japan), 210 U/mL DNase 1 (Fujifilm Wako Chemicals), and 3 mg/mL dispase 2 (Fujifilm Wako Chemicals). Following digestion, the cell suspension was agitated vigorously 20 times, filtered through a 40 μm cell strainer (Greiner Japan, Tokyo, Japan), and centrifuged (500× *g*, 10 min, 4 °C). The resulting cell pellet (mononuclear lamina propria (LP) cells) was resuspended in PBS containing 1% bovine serum albumin (BSA) and anti-CD16/32 antibody (BioLegend) for fragment crystallizable (Fc)-region blocking; the cells were subsequently stained for 1 h with fluorescein isothiocyanate (FITC)-conjugated anti-mouse IgA (BD Pharmingen, San Diego, CA, USA) and phycoerythrin (PE)-cyanine5 (Cy5)-conjugated anti-mouse B220 (CD45R) antibody (TONBO Biosciences, San Diego, CA, USA). The stained cells then were analyzed by flow cytometry using a Gallios flow cytometer (Beckman Coulter, Brea, CA, USA). IgA-PCs were defined as IgA^+^/CD45R^low^ cells [30]. The proportion of IgA-PCs among the colonic mononuclear LP cells was calculated.

### 2.6. Quantitative PCR Analysis

Total RNA was extracted from the colonic tissues stored in ISOGEN II; extraction was performed according to the manufacturer’s protocol. cDNA was synthesized from 250 ng of total RNA using ReverTra Ace (Toyobo, Osaka, Japan). The resulting cDNA was mixed with primer pairs and GeneAce SYBR qPCR Mix *α* (Nippon Gene). Amplifications were performed with an AriaMx Real-Time PCR thermocycler (Agilent Technologies, Tokyo, Japan) using the following temperature profile: one cycle at 95 °C for 10 min, followed by 40 cycles of denaturation at 95 °C for 30 s and annealing at 60 °C for 60 s. Values for the sample quantification cycles were normalized by comparison to those obtained for the transcript encoding the housekeeping protein β-actin. Fold changes in expression, relative to the control group, were then calculated using the ∆∆Cq method. The primer pair sequences are provided in Table 3.

### 2.7. Quantification of SCFA Concentrations in Cecal Content

Each cecal content sample was dispersed in a solution of 12% (*v*/*v*) formic acid (Tokyo Chemical Industry, Tokyo, Japan) supplemented with 1 mM 2-ethylbutyrate (Tokyo Chemical Industry; internal standard). The mixture was incubated at room temperature for 30 min. After centrifugation (9000× *g*, 5 min, room temperature), the supernatant was injected into a gas chromatograph (GC14-A, Shimadzu, Kyoto, Japan) equipped with a capillary FFAP column (15 m × 0.53 mm i.d.; GL Science, Tokyo, Japan). A serially diluted mixture of acetate, propionate, and butyrate supplemented with an internal control was prepared and analyzed for construction of the standard curves. Helium was used as the carrier gas. The injector and detector temperatures were maintained at 180 and 200 °C, respectively. The column temperature was increased from 80 to 200 °C at 10 °C/min over 12 min. A D-2500 Chromato-integrator (HITACHI, Tokyo, Japan) was used for peak picking; standard curve construction; and the determination of acetate, propionate, and butyrate concentrations in the cecal content samples.

### 2.8. Statistical Analysis

Results are presented as mean ± SEM or median (interquartile range). All statistical analyses were conducted using Prism for Windows (version 7.00; GraphPad Software, San Diego, CA, USA). The Shapiro–Wilk test was applied to test for normality. For data that failed the normality test, differences among groups were evaluated using the Kruskal–Wallis test. For data that passed the normality test, equality of variance was tested using Bartlett’s test. Based on the results of Bartlett’s test, differences among groups were evaluated using a one-way analysis of variance (ANOVA) or the Kruskal–Wallis test. Where overall significance was returned, these tests were followed by post hoc multiple comparisons tests adjusted by the false discovery rate using the method of Benjamini and Hochberg. Associations between each factor were evaluated using Pearson’s correlation for normally distributed data and Spearman’s rank correlation for non-normally distributed data. All analyses were conducted as two-tailed tests. *p*-values < 0.05 were considered statistically significant.

## 3. Results

### 3.1. Body Weight and Feed Intake

The final body weight and average feed intake are shown in Table 4. There was no significant difference in the final body weight or average feed intake among the groups.

### 3.2. ICI at the Phylum and Genus Levels

Values for the ICI at the phylum and genus levels are shown in Table 5 and Table 6, respectively. At the phylum level, significant differences in the ICIs were observed between the control and intervention groups as follows: the ICI for Actinobacteriota in the CNN group was significantly lower than that in the control group; the ICIs for Bacillota in the CNN and βCD groups were significantly lower than that in the control group; and the ICIs for Verrucomicrobiota in the CNN, αCD, and βCD groups were significantly higher than that in the control group. No shared changes (compared to the control group) were observed among the intervention groups. However, among the intervention groups, significant differences in the ICI were observed as follows: the ICI for Actinobacteriota in the CNN group was significantly lower than that in the βCD group; the ICIs for Bacillota in the CNN and βCD groups were significantly lower than that in the γCD group; the ICIs for Deferribacterota in the CNN and βCD groups were significantly lower than those in the αCD and γCD groups; the ICIs for Verrucomicrobiota in the CNN, αCD, and βCD groups were significantly higher than that in the γCD group; and the ICI for Bacteroidota in the βCD group was significantly higher than that in the γCD group.

At the genus level, significant differences in the ICIs were observed between the control and intervention groups as follows: the ICIs for Akkermansia in the CNN, αCD, and βCD groups were significantly higher than that in the control group; the ICIs for Clostridia UCG-014 in all of the intervention groups were significantly higher than that in the control group; the ICIs for the Lachnospiraceae NK4A136 group and Tuzzerella in the CNN group were significantly lower than those in the control group; the ICIs for UC Lachnospiraceae in the CNN and βCD groups were significantly lower than that in the control group; the ICIs for Erysipelatoclostridium, the Eubacterium coprostanoligenes group, Lachnoclostridium, Lachnospiraceae GCA-900066575, UC Peptococcaceae, and RF39 in the βCD group were significantly higher than those in the control group; and the ICI for Oscillibacter in the γCD group was significantly higher than that in the control group.

Among the CNN and CD groups, significant differences in the ICIs were observed as follows: the ICI for the Lachnospiraceae NK4A136 group in the CNN group was significantly lower than those in all of the CD groups; the ICIs for Acetatifactor and Mucispirillum in the CNN group were significantly lower than those in the αCD and γCD groups; the ICIs for Colidextribacter and Tuzzerella in the CNN group were significantly lower than those in the βCD and γCD groups; the ICIs for the Eubacterium coprostanoligenes group, Lachnoclostridium, Lachnospiraceae GCA-900066575, and UC Oscillospiraceae in the CNN group were significantly lower than those in the βCD group; the ICI for UC Lachnospiraceae in the CNN group was significantly lower than that in the γCD group; and the ICI for UC Erysipelotrichaceae in the CNN group was significantly higher than that in the αCD group.

Among the CD groups, significant differences in the ICIs were observed as follows: the ICI for Escherichia-Shigella in the αCD group was significantly lower than that in the γCD group; the ICIs for Lachnospiraceae GCA-900066575 in the αCD and γCD groups were significantly lower than that in the βCD group; the ICI for Lachnospiraceae UCG-006 in the αCD group was significantly lower than those in the βCD and γCD groups; the ICI for RF39 in the αCD group was significantly lower than that in the βCD group; the ICIs for Acetatifactor in the αCD and γCD groups were significantly higher than that in the βCD group; and the ICI for UC Lachnospiraceae in the βCD group was significantly lower than that in the γCD group.

### 3.3. Fecal IgA Concentration, Proportion of IgA-Secreting PCs in Colonic LP, and Colonic Gene Expression of Class Switching Recombination-Related Genes

**Figure 1:**
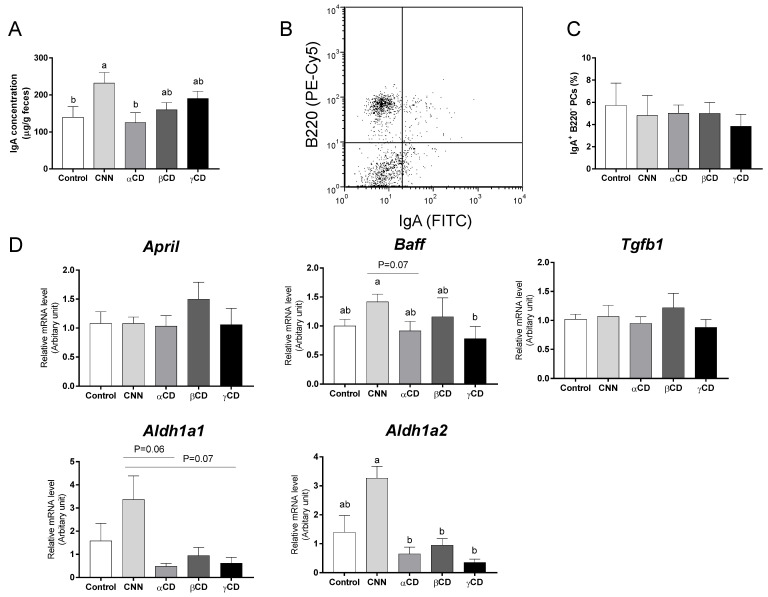
Quantification of fecal immunoglobulin A (IgA) concentration, proportion of colonic IgA-secreting plasma cells (IgA-PCs), and mRNA expression in the colon of IgA class-switching recombination-related genes. (**A**) The fecal IgA concentration. (**B**) Representative flow cytometric results for the detection of colonic IgA-PCs. (**C**) Proportions of IgA-PCs among the colonic mononuclear lamina propria (LP) cells. (**D**) Transcript levels of genes including *Aldh1a1*, *Aldh1a2*, *April*, *Baff*, and *Tgfb1*. Data are presented as mean ± SEM (*n* = 6). Following analysis of variance by Bartlett’s test, data were analyzed by two-tailed one-way ANOVA (equal variances) or Kruskal–Wallis test (unequal variances), and then by post hoc multiple comparisons tests, as needed. Values without a shared letter exhibited statistically significant differences (*p* < 0.05). Abbreviations: CNN: cyclic nigerosyl-1,6-nigerose; CD: cyclodextrin.

The fecal IgA concentration in the CNN group was significantly higher than those in the control and αCD groups (Figure 1A). There was no significant difference in the proportion of IgA-secreting PCs (IgA^+^ B220^−^) in the colonic LP (Figure 1B,C). Transcript levels of the genes encoding aldehyde dehydrogenase 1 family, member A1 (Aldh1a1), aldehyde dehydrogenase 1 family, member A2 (Aldh1a2), a proliferation-inducing ligand (April), B-cell-activating factor (Baff), and transforming growth factor beta 1 (Tgfb1) in the colon are shown in Figure 1D. There was no significant difference in the transcript levels of April and Tgfb1 among the groups. The CNN group exhibited a significant accumulation of Baff mRNA compared to the γCD group. The levels of Aldh1a1 transcript in the CNN group were nominally (but not significantly) higher than those in the αCD and γCD groups (*p* = 0.06 and *p* = 0.07, respectively). The levels of Aldh1a2 transcript in the CNN group were significantly higher than those in the αCD, βCD, and γCD groups, and nominally (but not significantly) higher than those in the control group (*p* = 0.07).

### 3.4. SCFA Concentrations in Cecal Content

**Figure 2:**
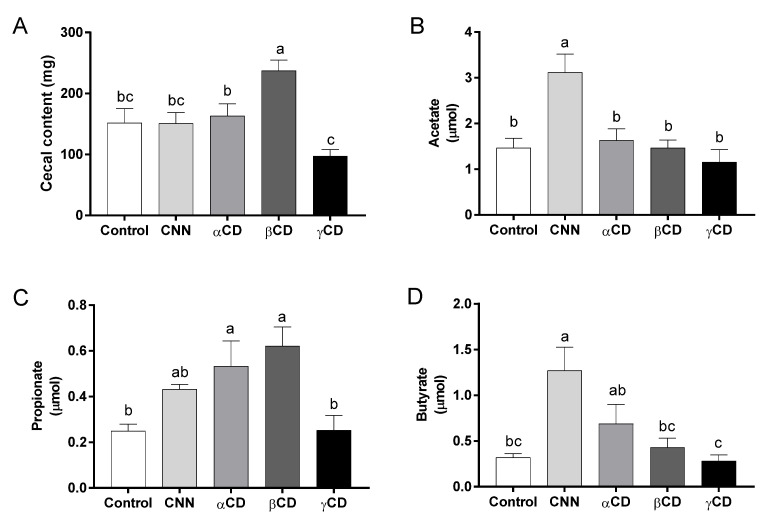
Amounts of short-chain fatty acids (SCFAs) in cecal content samples. (**A**) Cecal content weight. Amounts of (**B**) acetate, (**C**) propionate, and (**D**) butyrate in cecal content samples. Data are presented as mean ± SEM (*n* = 6). Following analysis of variance by Bartlett’s test, data were analyzed by two-tailed one-way ANOVA (equal variances) or Kruskal–Wallis test (unequal variances), and then by post hoc multiple comparisons tests, as needed. Values without a shared letter exhibited statistically significant differences (*p* < 0.05). Abbreviations: CNN: cyclic nigerosyl-1,6-nigerose; CD: cyclodextrin.

The cecal content weight and the acetate, propionate, and butyrate concentrations in the cecal content are shown in Figure 2. The cecal content weight was significantly higher in the βCD group than in the other groups (Figure 2A). The acetate concentration was significantly higher in the CNN group than in the other groups (Figure 2B). The propionate concentrations were significantly higher in the αCD and βCD groups than in the control and γCD groups (Figure 2C). The butyrate concentration was significantly higher in the CNN group than in the control, βCD, and γCD groups (Figure 2D).

### 3.5. RA in Total Fecal Bacteria and the Correlation of This Parameter with ICI

Values for the RAs in total fecal bacteria at the phylum and genus levels are shown in Table 7 and Table 8, respectively. At the phylum level, significant differences in the RAs were observed between the control and intervention groups as follows: the RA for Bacillota in the CNN group was significantly lower than that in the control group and the RAs for Bacteroidota and Verrucomicrobiota in the CNN group were significantly higher than those in the control group. No shared changes (compared to the control group) were observed within the intervention groups. Among the intervention groups, significant differences in the RAs were observed as follows: the RA for Actinobacteriota in the CNN group was significantly higher than that in the αCD group and the RA for Verrucomicrobiota in the CNN group was significantly higher than those in the βCD and γCD groups.

At the genus level, significant differences in the RAs were observed between the control and intervention groups as follows: the RAs for Acetatifactor in the CNN, αCD, and βCD groups were significantly lower than that in the control group; the RAs for Erysipelatoclostridium in the CNN, βCD, and γCD groups were significantly lower than that in the control group; the RAs for Mucispirillum in the βCD and γCD groups were significantly lower than that in the control group; the RAs for Peptococcus in the αCD and βCD groups were significantly lower than that in the control group; the RAs for Tuzzerella in the CNN and αCD groups were significantly lower than that in the control group; the RAs for Akkermansia, Bacteroides, and Clostridia UCG-014 in the CNN group were significantly higher than those in the control group; the RAs for Colidextribacter, Lachnospiraceae GCA-900066575, Lachnospiraceae NK4A136 group, UC Lachnospiraceae, and Lactococcus in the CNN group were significantly lower than those in the control group; the RAs for UC Erysipelotrichaceae and UC Peptococcaceae in the βCD group were significantly lower than that in the control group; and the RA for Ruminococcaceae CAG-352 in the γCD group was significantly lower than that in the control group.

Among the CNN and CD groups, significant differences in the RAs were observed as follows: the RAs for Lachnospiraceae GCA-900066575 and the Lachnospiraceae NK4A136 group in the CNN group were significantly lower than those in all of the CD groups; the RAs for Colidextribacter, Lachnoclostridium, UC Lachnospiraceae, and Tuzzerella in the CNN group were significantly lower than those in the βCD and γCD groups; the RA for Lactococcus in the CNN group was significantly lower than that in the γCD group; the RAs for Bacteroides and RF39 in the CNN group were significantly higher than those in all of the CD groups; the RAs for Akkermansia and Clostridia UCG-014 in the CNN group were significantly higher than those in the βCD and γCD groups; and the RA for Lachnospiraceae UCG-006 in the CNN group was significantly higher than that in the αCD group.

Among the CD groups, significant differences in the RAs were observed as follows: the RAs for Lachnoclostridium and Tuzzerella in the αCD group were significantly lower than those in the βCD and γCD groups; the RAs for Erysipelatoclostridium and Ruminococcaceae CAG-352 in the αCD group were significantly higher than those in the βCD and γCD groups.

**Figure 3:**
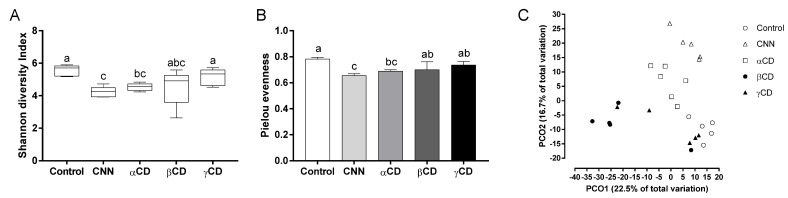
Analysis of alpha- and beta-diversity. Alpha-diversity analysis included (**A**) Shannon diversity and (**B**) Pielou’s evenness indices of the bacterial community. (**C**) Beta-diversity analysis using principal coordinate analysis based on unweighted UniFrac distance. Data are presented as median (interquartile range) (*n* = 6). The Shapiro–Wilk test was applied to test for normality. For data that failed the normality test, differences among groups were evaluated using the two-tailed Kruskal–Wallis test. For data that passed the normality test, equality of variance was tested using Bartlett’s test. Based on the results of Bartlett’s test, differences among groups were evaluated using two-tailed one-way ANOVA (equal variances) or Kruskal–Wallis test (unequal variances), and then by post hoc multiple comparisons tests, as needed. Values without a shared letter exhibited statistically significant differences (*p* < 0.05). Abbreviations: CNN: cyclic nigerosyl-1,6-nigerose; CD: cyclodextrin.

The CNN and αCD groups exhibited significantly lower Shannon diversity and Pielou’s evenness indices than the control group (Figure 3A,B). A principal coordinate analysis plot showed that the gut bacterial composition in the CNN, αCD, and βCD groups differed from that of the control group, and the composition differed among the intervention groups (Figure 3C).

**Figure 4:**
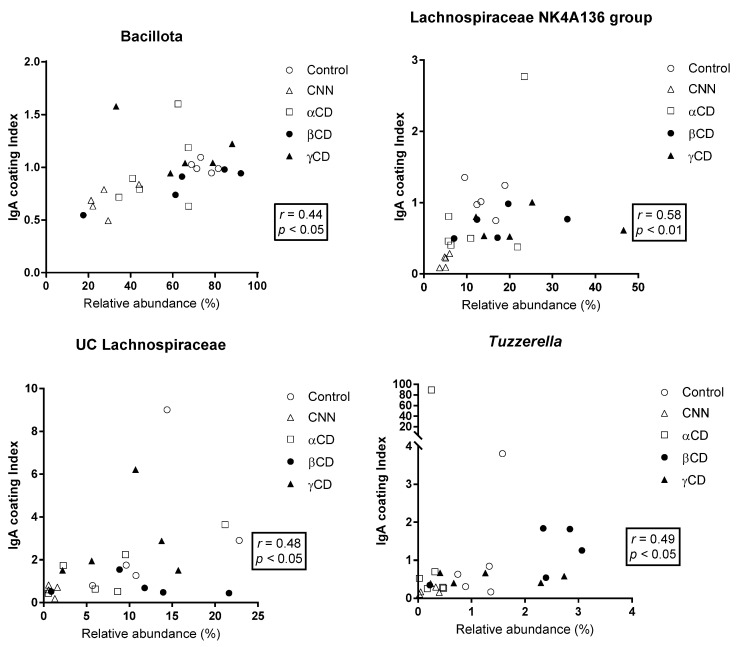
Correlation between the relative abundance (RA) of total bacteria and the immunoglobulin A (IgA) coating index (ICI). The Shapiro–Wilk test was applied to test for normality. For data that passed the normality test, correlations were assessed using Pearson’s correlation test. For data that failed the normality test, correlations were assessed using Spearman’s correlation test. The phyla and genera that exhibited significant correlations are shown. Abbreviations: CNN: cyclic nigerosyl-1,6-nigerose; CD: cyclodextrin.

The correlations between the ICI and RA are shown in Figure 4. At the phylum level, RA was significantly and positively correlated with the ICI in Bacillota. In the other phyla, no significant correlations were observed. At the genus level, a significant and positive correlation between the ICI and RA was observed in the Lachnospiraceae NK4A136 group, UC Lachnospiraceae, and Tuzzerella.

## 4. Discussion

In the present study, we sought to confirm whether the intake of cyclic oligosaccharides (including CNN, αCD, βCD, and γCD) affects the IgA coating profile of commensal gut bacteria, a factor known to contribute to the cyclic oligosaccharide-induced alteration in the gut bacterial composition. We observed that intake of each of the tested cyclic oligosaccharides alters gut bacterial composition, consistent with the results of previous studies [10,13], while also resulting in changes in the ICI at both the phylum and genus levels. At the phylum level, the ICI for Bacillota was significantly and positively correlated with the RA for Bacillota in total fecal bacteria; in contrast, no significant correlations between ICI and RA were observed in other phyla. At the genus level, a significant positive correlation between the ICI and RA was observed in the Lachnospiraceae NK4A136 group and UC Lachnospiraceae. These observations are consistent with a previous report indicating that Bacillota and Lachnospiraceae are major targets of IgA in healthy human feces, and that these bacteria are under-represented in the feces from patients deficient for IgA (compared to feces from healthy human donors) [31]. IgA is highly glycosylated in the hinge region, secretory component, and J chain, facilitating non-canonical glycan-mediated binding to commensal gut bacteria [32,33]. Furthermore, Briliūtė et al. showed that many mutualistic gut *Bacteroides* spp. utilize the complex N-glycans harbored by IgA as a nutrient carbon source [34]. Although the nutritional impact of IgA on Bacillota and Lachnospiraceae remains unclear at this time, the positive correlations (observed in the present work) between the ICI and RA suggest that cyclic oligosaccharide-induced changes in the IgA coating profile for Bacillota and a subset of Lachnospiraceae may influence the growth and colonization by these microorganisms. In addition, a significant positive correlation between the ICI and RA also was observed in *Tuzzerella*. This genus has been implicated in the development of non-alcoholic steatohepatitis (NASH) [35] and of polystyrene microplastics (MPs)-induced colonic and hepatic inflammation [36]. Cao et al. demonstrated that the abundance of *Tuzzerella* in the gut increased in diet-induced NASH model mice; those authors further showed that treatment with naringenin, a flavonoid, alleviated NASH symptoms with a concomitant decrease in *Tuzzerella* abundance [35]. Separately, Zhang et al. demonstrated that mice orally administered with MPs for 4 weeks exhibited colonic and hepatic inflammation along with an increase in the RA for *Tuzzerella* in the feces. Those researchers also showed that MPs-induced colonic and hepatic inflammation, as well as the increase in *Tuzzerella* abundance, were ameliorated by the oral administration of epigallocatechin-3-gallate [36]. In the present study, the CNN group exhibited significantly lower ICI and RA for *Tuzzerella* than did the control, βCD, and γCD groups, suggesting that a CNN-induced decrease in the IgA coating of *Tuzzerella* may contribute to a decrease in the RA of this genus. Considered together, these results suggest that consumption of food materials such as CNN (leading to decreased IgA coating of *Tuzzerella*) may alleviate NASH and MPs-induced colonic and hepatic inflammation via decreases in the gut abundance of *Tuzzerella*.

At the same time, we were unable to detect any phyla or genera demonstrating significant negative correlations between the ICI and RA. Previous studies have proposed that IgA may regulate bacterial colonization, both positively and negatively, through the prevention or promotion of the exclusion of select bacteria depending on the bacterial growth rate and the size of bacterial aggregates in the gut [32,37]. Our observations suggest that cyclic oligosaccharide-induced changes in the IgA coating of gut bacteria may not result in the exclusion of the IgA-coated bacteria.

Gut IgA secretion is affected by several cytokines (including BAFF) and retinoic acid (a mediator produced by aldehyde dehydrogenase 1-family members A1 (ALDH1a1) and ALDH1a2), factors that are known to contribute to IgA class-switching recombination (CSR) [38,39,40,41]. Isobe et al. reported that butyrate induces the differentiation of T cell-independent IgA-secreting PCs, an effect that may be mediated through the increased accumulation of the *Aldh1a2* transcript in dendritic cells. Consistent with previous reports, we observed that the administration of CNN significantly increases (compared to CDs) fecal IgA concentrations while also increasing cecal butyrate concentrations and *Baff*, *Aldh1a1*, and *Aldh1a2* transcript levels. Nonetheless, cyclic oligosaccharide-induced alterations in the ICI for Bacillota, the Lachnospiraceae NK4A136 group, UC Lachnospiraceae, and *Tuzzerella* did not depend on the fecal IgA concentration, suggesting that the amount of IgA secreted into the gut lumen may not be a determining factor in the changes observed in the IgA coating of these bacteria.

The CNN-fed group exhibited significantly lower ICIs for the Lachnospiraceae NK4A136 group and *Tuzzerella* (compared to control animals), while the ICIs in the CD-fed groups were comparable to those in the control animals. Additionally, animals administered CNN demonstrated significant increases in the acetate concentration of the cecal content compared to other groups. Furthermore, we showed that mice receiving CNN exhibited significant increases (compared to control animals) in the RA in the total fecal bacteria of the genus *Bacteroides*, a primary producer of acetate [42]. Notably, Takeuchi et al. reported that the reactivity of gut IgA to commensal bacteria is regulated by acetate. Specifically, those researchers showed that a diet containing water-soluble cellulose acetate is associated with a selective increase in gut acetate levels; this change induces, in the lower gastrointestinal tract of mice, increased secretion (compared to that observed with a control diet) of IgA with reactivity to gut bacteria, a change that is mediated via a T-cell-dependent pathway [28]. Based on the literature and our observations, we conjecture that *Bacteroides* spp. may utilize CNN as a substrate via hydrolysis by a cycloalternan-degrading enzyme [43]; the resulting increase in the cecal acetate level is expected to contribute to changes in IgA reactivity to specific gut bacteria such as the Lachnospiraceae NK4A136 group and *Tuzzerella*. The glycosidic linkage type differs between CNN (alternating α-1,3/1,6-linkages) and CDs (α-1,4-linkages). Specific glycosidic linkages in CNN may contribute to the decreases in ICI observed for the Lachnospiraceae NK4A136 group and *Tuzzerella*.

Unlike animals maintained on other oligosaccharide-supplemented diets, only the βCD-fed group demonstrated significant increases in ICI values (compared to controls) for several genera, including *Erysipelatoclostridium*, the *Eubacterium coprostanoligenes* group, *Lachnoclostridium*, Lachnospiraceae GCA-900066575, UC Peptococcaceae, and RF39. These effects in the βCD groups were observed despite the lack of a significant difference in the cecal acetate concentration or in the RA of these genera in total fecal bacteria when comparing the control and βCD-fed groups. We infer that βCD intake may promote the IgA coating of these bacteria through a mechanism other than an acetate-induced pathway or an increase in specific IgA response to these genera in immune inductive sites, such as Peyer’s patches and colonic patches. Notably, the water solubility of βCD (18.5 g/L at 25 °C) is lower than that of CNN (461 g/L at 20 °C), αCD (145 g/L at 25 °C), and γCD (232 g/L at 25 °C) [44]. Nakajima et al. demonstrated that the water solubility of dietary fiber influences gut IgA secretion through regulation of the expression of activation-induced cytidine deaminase, a protein that is crucial for the class-switch recombination from IgM to IgA [45]. Although the exact underlying mechanisms remain to be elucidated, the low solubility of βCD may relate to the induction of the specific IgA coating profile observed in the βCD-fed group. The physiological significance of the βCD-induced increases in the ICI for these specific genera remains to be clarified. We previously demonstrated that mice maintained on HFD exhibit a significantly lower ICI for *Erysipelatoclostridium* (compared to control animals), and that the HFD-induced decrease in the ICI for *Erysipelatoclostridium* is counteracted by CNN administration [22]. Furthermore, the ICI for *Erysipelatoclostridium* is negatively correlated with serum endotoxin levels and the colonic level of the mRNA encoding tumor necrosis factor α, an inflammatory cytokine [22]. Additionally, previous studies have suggested that the IgA coating of gut bacteria plays a crucial role in the suppression of gut inflammation [18,46,47]. Together, these results indicate that βCD-induced promotion of the IgA coating of *Erysipelatoclostridium* potentially may alleviate HFD-induced gut inflammation. Further studies will be needed to verify whether βCD can exert effects similar to those seen with CNN in mice with HFD-induced obesity.

Nakajima et al. demonstrated that the IgA coating of commensal *Bacteroides thetaiotaomicron* increases the expression of loci encoding polysaccharide utilization proteins, resulting in changes in the bacterial community and fermentation profiles, as well as increases in the relative abundance of the phylum Bacillota and of the cecal butyrate concentration [48]. That work implies that the IgA coating of specific gut bacteria indirectly modifies the composition of symbiont populations. In the present study, we observed that the intake of cyclic oligosaccharides significantly altered the RA of many genera, independent of the ICI profile. Cyclic oligosaccharide-induced changes in the IgA coating of specific gut bacteria may indirectly alter the RA of the symbiont populations.

We acknowledge that our study has some limitations. Notably, the results were obtained in a mouse model. Our study also has possible sources of bias, including previous evidence of uneven detection of certain 16S rRNA genes as a result of selective effects during bacterial DNA extraction and PCR amplification.

## 5. Conclusions

To our knowledge, the current study is the first to show that the oral administration of cyclic oligosaccharides, including CNN, αCD, βCD, and γCD, distinctly alters the IgA coating profile of commensal gut bacteria in mice. Among the surveyed gut bacteria, Bacillota, the Lachnospiraceae NK4A136 group, UC Lachnospiraceae, and *Tuzzerella* demonstrated significant positive correlations between the ICI and RA; other phyla and genera did not exhibit significant correlations between these parameters. Our observations suggest that cyclic oligosaccharide-induced modulation of the IgA coating of gut bacteria may contribute (in part) to changes in the bacterial community structure. Furthermore, our study indicates that the evaluation of the modulatory effect of food materials on the IgA coating profile of commensal gut bacteria may provide information regarding methods for regulating the abundance of specific gut bacteria in humans.

## Figures and Tables

**Table 1:** Composition of experimental diets.

Ingredients (g/kg Diet)	Control Diet	Cyclic-Oligosaccharide-Supplemented Diet
α- Maize starch	529.5	479.5
Casein	200	200
Sucrose	100	100
Cellulose	50	50
Soybean oil	70	70
AIN-93 Mineral mix	35	35
AIN-93 Vitamin mix	10	10
L-Cystine	3	3
Choline bitartrate	2.5	2.5
Cyclic-oligosaccharide (CNN, αCD, βCD, or γCD)	-	50

Abbreviations: CNN: cyclic nigerosyl-1,6-nigerose; CD: cyclodextrin.

**Table 2:** Properties of cyclic oligosaccharides.

	Chain Length	Glycosidic Linkage Type	Water Solubility
Cyclic nigerosyl-1,6-nigerose (CNN)	4	α-1,3 and α-1,6	Soluble
α-Cyclodextrin (αCD)	6	α-1,4	Soluble
β-Cyclodextrin (βCD)	7	α-1,4	Insoluble
γ-Cyclodextrin (γCD)	8	α-1,4	Soluble

**Table 3:** Primer sets used for quantitative PCR.

Target gene		Sequence
*Aldh1a1*	Forward	CTGGCTACAATGGAGGCACTCA
	Reverse	AGTGAAAATGTCTCCATCACTTGGT
*Aldh1a2*	Forward	TGGTATCCTCCGCAATGCAA
	Reverse	TCCCGTAAGCCAAACTCACC
*April*	Forward	TGGAAGGATGGGGCGAAATC
	Reverse	ACGTCAGAGTCTGCCTTGGA
*Baff*	Forward	CGACACGCCGACTATACGAA
	Reverse	GCCTGTTTGCCTCACCACTA
*Tgfb1*	Forward	GCCTGAGTGGCTGTCTTTTG
	Reverse	GTGAGCGCTGAATCGAAAGC

Abbreviations: *Aldh1a1*: aldehyde dehydrogenase 1 family, member A1, *Aldh1a2*: aldehyde dehydrogenase 1 family, member A2, *April*: a proliferation-inducing ligand, *Baff*: B-cell-activating factor, *Tgfb1*: transforming growth factor beta 1.

**Table 4:** Final body weight and average feed intake.

	Control	CNN	αCD	βCD	γCD
Final body weight (g)	36.7 ± 1.5	38.1 ± 1.0	38.4 ± 0.6	35.9 ± 0.5	36.7 ± 1.5
Average feed intake (g/mouse/day)	4.3 ± 0.3	5.2 ± 1.0	5.3 ± 1.6	4.2 ± 0.7	3.9 ± 0.4

Values are given as means ± SEMs (*n* = 6 per group). Abbreviations: CNN: cyclic nigerosyl-1,6-nigerose; CD: cyclodextrin.

**Table 5:** IgA coating index at phylum level ^1,2^.

	Control	CNN	αCD	βCD	γCD
Actinobacteriota	0.63 (0.50–5.55) ^a^	0.11 (0.09–0.13) ^b^	0.32 (0.07–1.01) ^ab^	0.68 (0.52–1.22) ^a^	0 (0–4.69) ^ab^
Bacillota	0.99 (0.97–1.06) ^a^	0.69 (0.56–0.81) ^c^	0.84 (0.70–1.29) ^abc^	0.91 (0.64–0.96) ^bc^	1.04 (0.99–1.40) ^a^
Bacteroidota	0.29 (0.29–2.16) ^ab^	1.10 (0.76–1.56) ^ab^	1.77 (0.29–1.91) ^ab^	2.33 (0.94–5.00) ^a^	0.50 (0.35–0.84) ^b^
Deferribacterota	8.67 (2.43–10.91) ^ab^	0.85 (0.40–2.76) ^b^	21.00 (5.67–101.15) ^a^	0 ^c^	13.60 (4.64–24.72) ^a^
Pseudomonadota	15.54 (4.03–61.54)	69.92 (36.01–93.67)	6.91 (4.11–38.05)	12.89 (11.62–30.35)	21.37 (14.31–33.44)
Verrucomicrobiota	0.43 (0.29–0.52) ^b^	1.30 (0.67–4.77) ^a^	1.95 (1.42–3.43) ^a^	3.64 (1.25–14.10) ^a^	0.61 (0.37–1.99) ^b^

^1^ Values are given as median (interquartile range) (*n* = 6 per group). Values without a common letter are statistically significantly different (*p* < 0.05). ^2^ One-way ANOVA or Kruskal Wallis test followed by post-hoc test was conducted. Abbreviations: CNN: cyclic nigerosyl-1,6-nigerose; CD: cyclodextrin.

**Table 6:** IgA coating index at genus level ^1,2^.

	Control	CNN	αCD	βCD	γCD
*Acetatifactor*	0.12 (0.11–0.40) ^ab^	0 (0–0.30) ^b^	0.48 (0.29–17.95) ^a^	0 (0–0.35) ^b^	0.61 (0.37–1.21) ^a^
*Akkermansia*	0.43 (0.29–0.52) ^c^	1.30 (0.67–4.77) ^ab^	1.95 (1.42–3.43) ^ab^	3.64 (1.25–14.11) ^a^	0.61 (0.37–2.00) ^bc^
*Bacteroides*	1.75 (1.42–2.68)	1.75 (1.30–3.09)	2.26 (0.65–6.20)	1.12 (0.58–2.46)	2.31 (0.94–4.72)
Clostridia UCG-014	0.47 (0.19–0.56) ^b^	1.18 (0.93–2.01) ^a^	1.31 (0.54–5.20) ^a^	2.00 (0.77–8.87) ^a^	0.96 (0.77–1.50) ^a^
*Colidextribacter*	0.25 (0.14–0.52) ^ab^	0.52 (0.13–0.69) ^b^	0.38 (0.35–2.19) ^ab^	0.99 (0.51–1.49) ^a^	0.63 (0.52–1.00) ^a^
*Erysipelatoclostridium*	3.46 (2.16–8.72) ^b^	8.68 (2.88–16.09) ^ab^	8.44 (3.56–10.22) ^ab^	18.49 (7.09–29.84) ^a^	7.02 (4.75–9.85) ^ab^
UC Erysipelotrichaceae	0.50 (0.13–0.93) ^ab^	2.40 (0.81–56.46) ^a^	0.32 (0.24–0.61) ^b^	0.70 (0.59–8.74) ^ab^	4.95 (0.70–11.21) ^ab^
*Escherichia-Shigella*	162.80 (17.22–1326.00) ^ab^	81.45 (48.37–116.90) ^ab^	21.06 (10.71–95.57) ^b^	28.32 (21.33–271.20) ^ab^	173.60 (108.00–470.30) ^a^
*Eubacterium coprostanoligenes* group	0.23 (0.21–1.22) ^b^	0.50 (0.36–1.54) ^b^	1.72 (0.86–3.13) ^ab^	8.82 (2.06–24.68) ^a^	1.46 (0.81–5.45) ^ab^
*Lachnoclostridium*	0.20 (0.17–0.39) ^b^	0.07 (0–0.47) ^b^	0.29 (0.20–0.46) ^ab^	0.59 (0.36–0.76) ^a^	0.26 (0.21–0.30) ^ab^
Lachnospiraceae GCA-900066575	0.17 (0.06–0.27) ^b^	0.26 (0.17–0.27) ^b^	0.23 (0.19–0.25) ^b^	0.46 (0.40–1.22) ^a^	0.20 (0.17–0.68) ^b^
Lachnospiraceae FCS020 group	0.60 (0.39–1.09)	0.20 (0.02–0.48)	0.46 (0–0.74)	0.66 (0.37–0.92)	0.46 (0.23–0.94)
Lachnospiraceae NK4A136 group	1.01 (0.86–1.30) ^a^	0.22 (0.09–0.27) ^b^	0.48 (0.40–1.30) ^a^	0.76 (0.51–0.88) ^a^	0.61 (0.53–0.91) ^a^
Lachnospiraceae UCG-006	0.13 (0.04–0.16) ^ab^	0.16 (0.06–0.24) ^ab^	0.10 (0–0.13) ^b^	0.44 (0.09–0.82) ^a^	0.30 (0.16–0.42) ^a^
UC Lachnospiraceae	1.75 (1.04–5.95) ^a^	0.59 (0.19–0.77) ^b^	1.18 (0.50–2.59) ^ab^	0.52 (0.47–1.12) ^b^	1.94 (1.51–4.54) ^a^
*Lactococcus*	1.70 (0.60–2.00) ^a^	0.87 (0.61–1.33) ^ab^	0.87 (0.58–0.95) ^b^	0.87 (0.65–1.40) ^ab^	0.82 (0.76–1.17) ^ab^
*Mucispirillum*	8.67 (2.43–10.91) ^ab^	1.18 (0.81–3.38) ^b^	30.73 (9.42–130.60) ^a^	ND	13.60 (4.65–24.73) ^a^
*Oscillibacter*	0.33 (0.25–0.57) ^b^	0.35 (0.20–0.64) ^b^	0.43 (0.38–0.83) ^b^	0.73 (0.47–0.90) ^ab^	0.84 (0.74–1.67) ^a^
UC Oscillospiraceae	0.58 (0.44–0.89) ^ab^	0 (0–0.19) ^b^	0.45 (0–0.50) ^ab^	1.99 (0.30–2.42) ^a^	0.90 (0–220.60) ^ab^
*Peptococcus*	0.13 (0.11–0.62)	0	0.45 (0.23–16.89)	5.96 (0.37–11.55)	0.45 (0.21–4.12)
UC Peptococcaceae	0.25 (0.12–0.63) ^b^	0.48 (0.12–1.74) ^ab^	0.35 (0.27–0.78) ^ab^	1.73 (0.60–3.89) ^a^	1.43 (0.16–3.20) ^ab^
RF39	0.55 (0.43–1.40) ^bc^	0.93 (0.76–1.29) ^abc^	0.37 (0.24–0.69) ^c^	3.10 (1.40–3.53) ^a^	2.96 (0.62–12.98) ^ab^
Ruminococcaceae CAG-352	0.99 (0.78–3.33)	1.70 (0.80–2.03)	0.49 (0.39–19.88)	11.35 (0.19–63.95)	17.57 (3.85–50.74)
*Tuzzerella*	0.63 (0.24–2.33) ^a^	0.17 (0.13–0.35) ^b^	0.41 (0.27–22.79) ^ab^	1.25 (0.45–1.83) ^a^	0.58 (0.40–0.67) ^a^

^1^ Values are given as median (interquartile range) (*n* = 6 per group). Values without a common letter are statistically significantly different (*p* < 0.05). ^2^ One-way ANOVA or Kruskal Wallis test followed by post-hoc test was conducted. Abbreviations: CNN: cyclic nigerosyl-1,6-nigerose; CD: cyclodextrin.

**Table 7:** Relative abundance in total gut bacteria at phylum level ^1,2^.

	Control	CNN	αCD	βCD	γCD
Actinobacteriota	0.01 (0–0.05) ^ab^	0.07 (0.04–0.11) ^a^	ND ^b^	0.00 (0–0.03) ^ab^	0 (0–0.07) ^ab^
Bacillota	73.19 (70.06–79.92) ^a^	27.39 (21.69–36.66) ^b^	53.34 (39.30–67.33) ^ab^	64.34 (39.40–88.34) ^ab^	65.80 (46.00–83.52) ^ab^
Bacteroidota	23.89 (18.44–25.62) ^b^	59.58 (47.19–64.25) ^a^	37.34 (28.47–45.82) ^ab^	30.54 (11.38–56.24) ^ab^	33.22 (13.41–52.27) ^ab^
Deferribacterota	0.32 (0.25–1.70)	0.05 (0.01–0.34)	0.06 (0–0.37)	0 (0–0.18)	0.07 (0–0.18)
Pseudomonadota	0.28 (0.02–1.82)	0.08 (0.04–3.05)	0.11 (0.09–0.34)	0.28 (0.18–2.46)	0.51 (0.18–2.87)
Verrucomicrobiota	1.13 (0.04–2.67) ^b^	13.49 (9.29–16.99) ^a^	8.10 (3.65–15.98) ^ab^	0.09 (0–4.16) ^b^	0.18 (0.16–1.44) ^b^

^1^ Values are given as median (interquartile range) (*n* = 6 per group). Values without a common letter are statistically significantly different (*p* < 0.05). ^2^ One-way ANOVA or Kruskal Wallis test followed by post-hoc test was conducted. Abbreviations: CNN: cyclic nigerosyl-1,6-nigerose; CD: cyclodextrin.

**Table 8:** Relative abundance in total gut bacteria at genus level ^1,2^.

	Control	CNN	αCD	βCD	γCD
*Acetatifactor*	2.22 (0.56–2.47) ^a^	0.05 (0–0.14) ^b^	0.17 (0–0.85) ^b^	0.07 (0–0.26) ^b^	0.70 (0.05–0.79) ^ab^
*Akkermansia*	1.13 (0.04–2.67) ^b^	13.49 (9.29–19.99) ^a^	8.10 (3.65–15.98) ^ab^	0.09 (0–4.16) ^b^	0.18 (0.16–1.44) ^b^
*Bacteroides*	5.40 (1.22–6.82) ^b^	30.26 (22.97–37.88) ^a^	11.06 (5.92–19.73) ^b^	9.89 (8.24–18.88) ^b^	6.44 (1.62–16.78) ^b^
Clostridia_UCG-014	0.30 (0.02–0.69) ^b^	4.75 (3.42–9.59) ^a^	3.91 (0.13–13.24) ^ab^	0.02 (0–3.87) ^b^	0.36 (0.04–0.60) ^b^
*Colidextribacter*	3.60 (2.20–5.50) ^a^	0.81 (0.53–1.13) ^b^	1.55 (0.94–2.32) ^ab^	4.72 (1.38–10.36) ^a^	2.32 (1.78–5.13) ^a^
*Erysipelatoclostridium*	4.99 (2.45–7.53) ^a^	0.34 (0.26–0.37) ^bc^	0.47 (0.41–1.37) ^ab^	0.01 (0–0.47) ^c^	0.18 (0.09–0.40) ^c^
UC Erysipelotrichaceae	0.20 (0.04–0.30) ^a^	0.04 (0.02–0.06) ^ab^	0.02 (0.02–0.09) ^ab^	0 (0–0.01) ^b^	0.03 (0–0.05) ^ab^
*Escherichia-Shigella*	0.28 (0.02–1.82)	0.08 (0.04–3.05)	0.11 (0.09–0.34)	0.28 (0.17–2.46)	0.51 (0.18–2.87)
Eubacterium coprostanoligenes group	0.61 (0.03–1.28)	0.04 (0.02–0.06)	0.24 (0.04–0.39)	0.21 (0.04–1.08)	0.27 (0.03–1.37)
*Lachnoclostridium*	0.54 (0.14–0.77) ^ab^	0.18 (0.01–0.19) ^b^	0.23 (0.15–0.52) ^b^	1.60 (0.65–2.28) ^a^	0.96 (0.63–2.37) ^a^
Lachnospiraceae GCA-900066575	0.44 (0.21–0.90) ^a^	0.08 (0.04–0.10) ^b^	0.50 (0.44–1.12) ^a^	0.42 (0.18–4.26) ^a^	0.41 (0.31–5.81) ^a^
Lachnospiraceae FCS020 group	0.09 (0.02–0.21)	0.08 (0.05–0.10)	0.07 (0.03–0.16)	0.10 (0.03–0.24)	0.17 (0.07–0.30)
Lachnospiraceae NK4A136 group	13.3 (10.93–17.83) ^a^	5.06 (4.24–5.52) ^b^	8.60 (5.76–22.22) ^a^	17.17 (9.70–26.57) ^a^	20.0 (13.09–35.92) ^a^
Lachnospiraceae UCG-006	0.07 (0.03–0.09) ^ab^	0.07 (0.01–0.13) ^a^	0 (0–0.02) ^b^	0.05 (0–0.11) ^ab^	0.08 (0.02–0.10) ^ab^
UC Lachnospiraceae	10.76 (7.66–18.61) ^a^	1.29 (0.57–1.45) ^b^	7.31 (1.84–12.44) ^ab^	11.77 (4.85–17.78) ^a^	10.71 (3.90–14.72) ^a^
*Lactococcus*	1.25 (0.74–1.63) ^a^	0.18 (0.11–0.21) ^b^	0.31 (0.15–0.63) ^ab^	0.51 (0.11–1.08) ^ab^	0.55 (0.39–1.07) ^a^
*Mucispirillum*	0.32 (0.25–1.70) ^a^	0.05 (0.01–0.34) ^ab^	0.06 (0–0.37) ^ab^	0 (0–0.18) ^b^	0.07 (0–0.18) ^b^
*Oscillibacter*	1.48 (1.28–2.37)	2.46 (0.75–3.05)	1.42 (0.89–5.21)	3.60 (0.94–10.13)	3.55 (2.05–6.77)
UC Oscillospiraceae	0.21 (0.08–0.30)	0.12 (0.02–0.12)	0.04 (0–0.26)	0.15 (0–1.69)	0.07 (0–0.76)
*Peptococcus*	0.16 (0.11–0.26) ^a^	0.11 (0.03–0.19) ^ab^	0.03 (0–0.12) ^b^	0 (0–0.03) ^b^	0.03 (0–0.19) ^ab^
UC Peptococcaceae	5.74 (2.89–10.58) ^a^	1.00 (0.92–1.78) ^ab^	1.54 (1.31–2.24) ^ab^	0.60 (0.28–3.52) ^b^	3.29 (0.65–5.67) ^ab^
RF39	0.16 (0.03–0.80) ^ab^	1.02 (0.29–2.20) ^a^	0.05 (0.03–0.06) ^b^	0 (0–0.06) ^b^	0.05 (0.03–0.15) ^b^
Ruminococcaceae CAG-352	4.16 (0.50–6.77) ^ab^	3.28 (2.07–3.99) ^abc^	9.95 (3.04–12.97) ^a^	0 (0–2.65) ^b^	0 (0–1.62) ^c^
*Tuzzerella*	1.33 (0.82–1.47) ^a^	0.23 (0.04–0.36) ^b^	0.28 (0.14–0.47) ^b^	2.39 (1.28–2.95) ^a^	1.26 (0.54–2.52) ^a^

^1^ Values are given as median (interquartile range) (*n* = 6 per group). Values without a common letter are statistically significantly different (*p* < 0.05). ^2^ One-way ANOVA or Kruskal Wallis test followed by post-hoc test was conducted. Abbreviations: CNN: cyclic nigerosyl-1,6-nigerose; CD: cyclodextrin.

## Data Availability

Data are contained within the article.
